# PKC-*ι* Regulates an Oncogenic Positive Feedback Loop Between the MAPK/JNK Signaling Pathway, c-Jun/AP-1 and TNF-*α* in Breast Cancer

**DOI:** 10.3390/ijms26157288

**Published:** 2025-07-28

**Authors:** Nuzhat Nowshin Oishee, Mahfuza Marzan, Abigail Oluwafisayo Olatunji, Khandker Mohammad Khalid, Abiral Hasib Shourav, Radwan Ebna Noor, Anna Kharitonova, Aaron Joshua Astalos, James W. Leahy, Mildred Acevedo-Duncan

**Affiliations:** Department of Chemistry, University of South Florida, 4202 E Fowler Ave, CHE 205, Tampa, FL 33620, USA; nuzhatn@usf.edu (N.N.O.); mmarzan@usf.edu (M.M.); abigailolatunji@usf.edu (A.O.O.); kmkhalid@usf.edu (K.M.K.); hasibshourav@usf.edu (A.H.S.); noor2@usf.edu (R.E.N.); kharitonova@usf.edu (A.K.); aronastalos@usf.edu (A.J.A.); jwleahy@usf.edu (J.W.L.)

**Keywords:** breast cancer, c-Jun, MAPK/JNK pathway, TNF-α, apoptosis

## Abstract

Breast cancer is the second most common cancer in the United States and consists of 30% of all new female cancer each year. PKC iota (PKC-ι) is a bonafide human oncogene and is overexpressed in many types of cancer, including breast cancer. This study explores the role of PKC-ι in regulating the transcription factor Jun proto-oncogene (c-Jun), pro-inflammatory cytokine Tumor Necrosis Factor-alpha (TNF-α), and the Mitogen-Activated Protein Kinase/Jun N-terminal kinase (MAPK/JNK) pathway, which also exhibits an oncogenic role in breast cancer. ICA-1S, a PKC-ι specific inhibitor, was used to inhibit PKC-ι to observe the subsequent effect on the levels of c-Jun, TNF-α, and the MAPK/JNK signaling pathway. To obtain the results, cell proliferation assay, Western blotting, co-immunoprecipitation, small interfering RNA (siRNA), immunofluorescence, flow cytometry, cycloheximide (CHX) chase assay, and reverse transcription quantitative PCR (RT-qPCR) techniques were implemented. ICA-1S significantly inhibited cell proliferation and induced apoptosis in both breast cancer cell lines. Treatment with ICA-1S and siRNA also reduced the expression levels of the MAPK/JNK pathway protein, c-Jun, and TNF-α in both cell lines. PKC-ι was also found to be strongly associated with c-Jun, via which it regulated the MAPK/JNK pathway. Additionally, ICA-1S was found to promote the degradation of c-Jun and decrease the mRNA levels of c-Jun. We concluded that PKC-ι plays a crucial role in regulating breast cancer, and the inhibition of PKC-ι by ICA-1S reduces breast cancer cell proliferation and induces apoptosis. Therefore, targeting PKC-ι as a potential therapeutic target in breast cancer could be a significant approach in breast cancer research.

## 1. Introduction

Breast cancer is the abnormal growth of breast cells, which often begins in the milk-producing ducts or lobules or other cells or tissue within the breast and is the second most common cancer in the United States [1]. Breast cancer consists of approximately 30% of all new female cancers each year. The World Health Organization forecasts that there will be approximately 2.5 million breast cancer deaths worldwide between 2020 and 2040 [1]. Treatment of breast cancer includes surgery in addition to supplementary treatment options such as chemotherapy, hormone therapy, or radiation therapy [1]. Chemotherapy might also be used before surgery, depending on the stage and type of breast cancer [1]. Although breast cancer treatment and management options have significantly improved over time, emphasizing targeted therapies and understanding specific therapeutic targets and the molecular mechanisms regulating it is crucial for achieving a more efficient approach for combating breast cancer.

One promising area of research in targeted therapy is the role of atypical protein kinase C (aPKC) isoforms. Understanding the function and regulation of aPKC isoforms could pave the way for innovative strategies to prevent cancer progression and enhance patient outcomes. Protein Kinase C is one of the most widely studied signaling kinases and is a family of serine/threonine kinases [2]. PKCs regulate various signaling pathways that control cell cycle progression, metastatic dissemination, and tumorigenesis [2,3]. Previous studies established that aPKC plays a significant role in maintaining the critical cellular functions essential in cancer and is overexpressed in many types of cancer, including breast cancer [3,4]. In a study, PKC-ι overexpression was detected in 88 of the 110 breast cancer cases, and the immunoreactivity of PKC-ι was strong in invasive ductal carcinoma [5].

c-Jun is the first cellular proto-oncogene and the most comprehensively studied member of the Activator Protein-1 (AP-1) transcription factor [6,7]. c-Jun-dependent transcription is activated by the phosphorylation of c-Jun at serine 63/73, which is stimulated by intracellular or extracellular signals comprising UV radiation, growth factors and transforming oncoproteins [8]. Studies have previously demonstrated the overexpression of c-Jun in human breast cancer and its involvement in cancer progression, cell proliferation, apoptosis, survival, tumorigenesis, and tissue morphogenesis [8]. In a clinical study, it has been demonstrated that out of 103 cases of invasive breast cancer, approximately 38% of invasive breast cancer cases showed a predominant nuclear expression of activated c-Jun [8]. Hence, the inhibition of c-Jun can act as a potential therapeutic target to prevent breast cancer progression.

c-Jun can also be activated through phosphorylation by JNK [9]. According to recent clinical research, increased JNK activity has been linked to a poor outcome in human breast cancer [9]. Activated JNK has also been implicated in cell migration and invasion in human breast cancer, helps in epithelial–mesenchymal transition (EMT), and helps the activity of AP-1 transcription complex, which is a dimeric complex that includes members of the JUN proto-oncogene, FOS proto-oncogene, Musculoaponeurotic fibrosarcoma (MAF), and Activating Transcription Factor (ATF) protein families [9,10]. Studies have also shown dysfunctional AP-1 in many types of cancer, including triple-negative breast cancer (TNBC) [11]. Previously, it was demonstrated that the blockade of AP-1 inhibited the growth of breast cancer cells both in vitro and in vivo [12]. In addition, since c-Jun is the predominant component of AP-1, the overexpression of c-Jun has been shown to drive cancer cell progression and survival, thereby increasing AP-1 activity, which in this case is considered oncogenic [11,12]. AP-1 is a transcriptional regulator of the pro-inflammatory cytokine, TNF-α, which also has been implicated in breast cancer progression and tumorigenesis [13].

Therefore, this study investigates the role of PKC-ι in breast cancer by utilizing a small molecule inhibitor 5-amino-1-((1R,2S,3S,4R)-2,3-dihydroxy-4-methylcyclopentyl)-1H-imidazole-4-carboxamide (ICA-1S) specific to PKC-ι. In this study, it is hypothesized that PKC-ι plays a crucial role in regulating a positive feedback loop between c-Jun/AP-1, TNF-α, and the MAPK/JNK pathway and examines the subsequent effect of PKC-ι inhibition by ICA-1S on this loop. Furthermore, this study explores if c-Jun is directly activated by PKC-ι, which is thought to regulate this signaling cascade. Targeting PKC-ι and understanding its role in the oncogenic MAPK/JNK signaling cascade could signify a novel breakthrough in breast cancer research.

## 2. Results

### 2.1. ICA-1S Inhibits the Cell Proliferation of Malignant Breast Cancer Cell Lines

**Figure 1:**
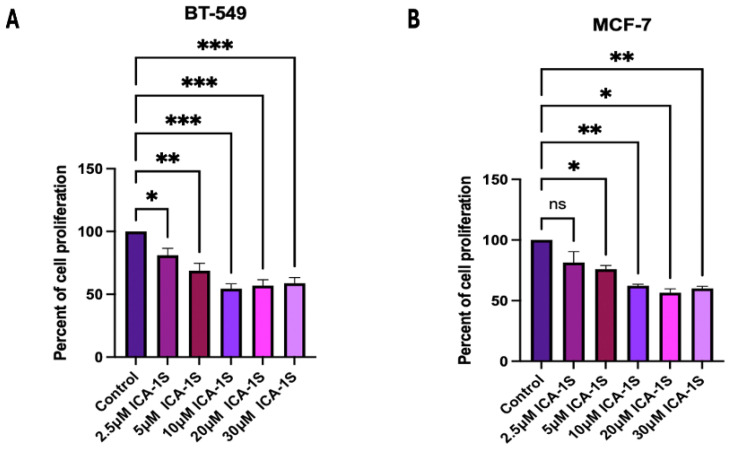
Dose response curve of ICA-1S in (**A**) BT-549 and (**B**) MCF-7 cell line. The results for the cell proliferation assay indicates ICA-1S has an inhibitory effect on the cell proliferation of breast cancer cell lines. The graph represents the mean ± Standard Error of Mean (SEM) of 3 independent experiments of each cell line. *** *p* < 0.001; ** *p* < 0.005, * *p* < 0.05, ns: not significant.

After a five-day treatment with various concentrations of ICA-1S, the results (Figure 1) indicated that ICA-1S significantly reduces the cell proliferation of both BT-549 and MCF-7 breast cancer cell lines. The $IC_{50}$ concentration, which is referred to the concentration at which 50% inhibition of the cell proliferation was obtained using ICA-1S, for the BT-549 cell line was at 10μM (45%, *p* < 0.001) and for the MCF-7 cell line was at 20μM (43%, *p* < 0.05).

### 2.2. ICA-1S Induce Apoptosis in Malignant Breast Cancer Cells

**Figure 2:**
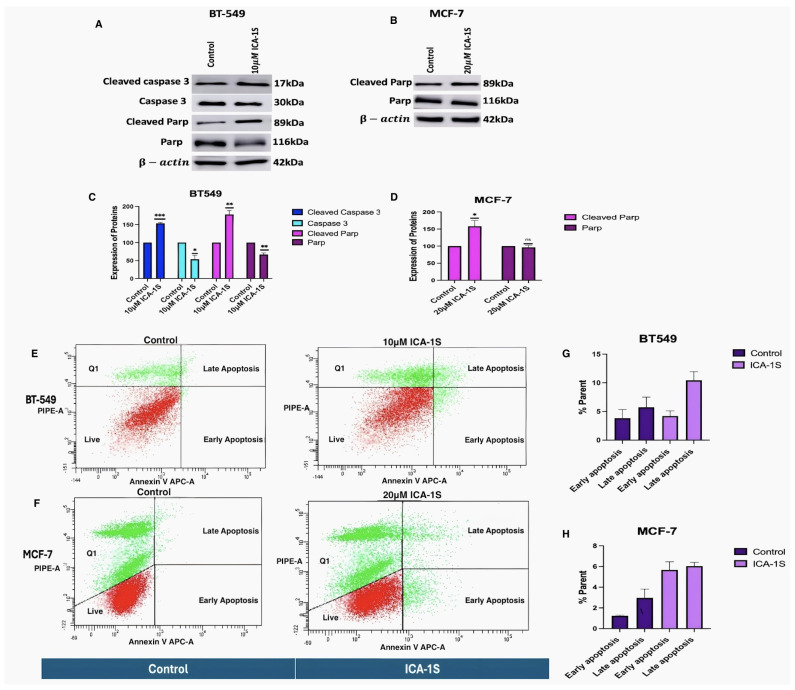
Treatment with ICA-1S induces apoptosis for both BT-549 and MCF-7 cells. (**A**–**D**) Representative Western blot images and the corresponding graph indicate that treatment with ICA-1S increases the levels of cleaved Caspase 3 and cleaved PARP, which are hallmark events in apoptosis. Expression levels were normalized to the corresponding loading controls such as β-actin and α-tubulin. (**E**–**H**) Flow cytometry analysis using Annexin V-APC/PI staining demonstrates an increase in both early and late apoptotic cell populations with ICA-1S treatment when compared to the control group (untreated group) as observed in the individual cell dot plots for both BT-549 and MCF-7 cells. The graph represents the mean ± Standard Error of Mean (SEM) of 3 independent experiments of each cell line. *** *p* < 0.001; ** *p* < 0.005, * *p* < 0.05. ns: not significant.

As there was a drastic change in the cell proliferation of the breast cancer cells with ICA-1S, it was then examined if this was due to apoptosis of the cells. To investigate whether cells were undergoing apoptosis after 5-day treatment with the IC_50_ concentration of ICA-1S, a Western blot analysis was performed to detect the expression of pro-apoptotic proteins. The results (Figure 2A–D) from the immunoblot analysis show that treatment with ICA-1S increases the levels of cleaved Caspase 3 (53%, *p* < 0.001) and cleaved Poly ADP-ribose polymerase (PARP) (78%, *p* < 0.005) for BT-549 cells. For MCF-7 cells, an increase in the levels of cleaved PARP (58%, *p* < 0.05) was observed. These results indicate that ICA-1S induces apoptosis in both BT-549 and MCF-7 cell lines.

Furthermore, the results (Figure 2E–H) for the flow cytometry analysis indicate that treatment with ICA-1S leads to increased early and late apoptosis compared to the control for BT-549 and MCF-7 cells.

### 2.3. Effect of ICA-1S on Atypical Protein Kinase C

**Figure 3:**
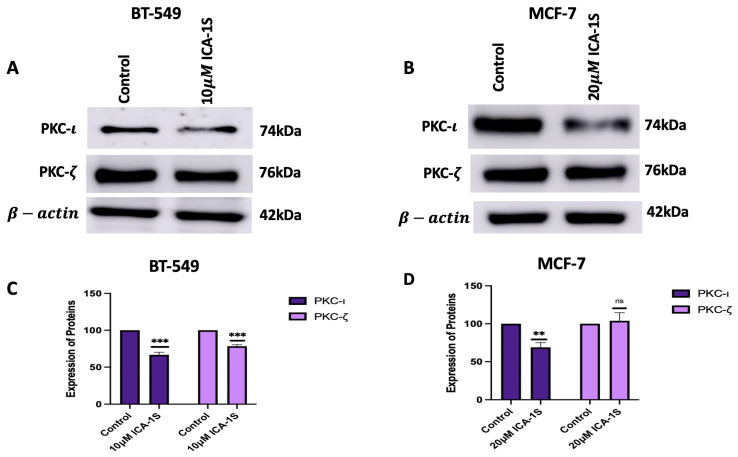
Representative (**A**,**C**) represents the Western blot images and its corresponding graphs for BT-549 cells and (**B**,**D**) represents the western blot images and its corresponding graph for MCF-7 cells. The results demonstrate the treatment with ICA-1S decreases the levels of PKC-ι in both BT-549 and MCF-7 cells, while the level of PKC-ζ was decreased in BT-549 cells but increased in MCF-7 cells. Expression levels were normalized to the corresponding loading controls such as β-actin and α-tubulin. The graph represents the Mean ± Standard Error of Mean (SEM) of 3 independent experiments of each cell line. *** *p* < 0.001; ** *p* < 0.005, ns: not significant.

As ICA-1S is a PKC-ι specific inhibitor, the effect of the IC_50_ concentration of ICA-1S on the levels of aPKCs was observed. The results (Figure 3) indicated that ICA-1S downregulates the level of both PKC-ι (33%, *p* < 0.001) and PKC-ζ (21%, *p* < 0.001) in BT-549 cells. On the contrary, for MCF-7 cells, ICA-1S downregulates the level of PKC-ι (31%, *p* < 0.005) but does not have any significant change in the levels of PKC-ζ.

### 2.4. ICA-1S Inhibits the Oncogenic Loop of the MAPK/JNK Pathway

**Figure 4:**
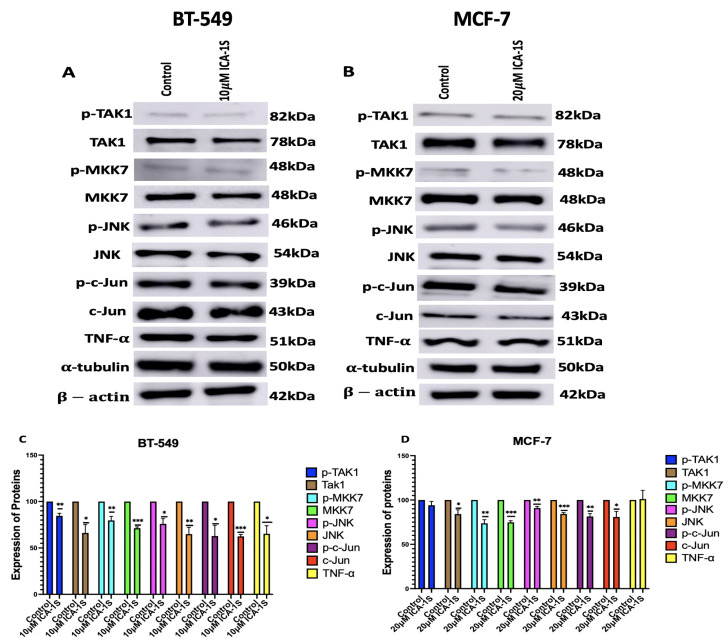
(**A**,**B**) Western blot images and (**C**,**D**) corresponding graphical representation demonstrating the effect of ICA-1S on the phosphorylation levels and the total protein level of the MAPK/JNK pathway proteins for BT-549 and MCF-7 cells. Expression levels were normalized to the corresponding loading controls such as β-actin and α-tubulin. The graph represents the Mean ± Standard Error of Mean (SEM) of 3 independent experiments of each cell line. *** *p* < 0.001; ** *p* < 0.005, * *p* < 0.05.

ICA-1S may have an inhibitory effect on the MAPK/JNK pathway proteins, as treatment with the IC_50_ concentration of ICA-1S has shown significant decrease in the MAPK/JNK pathway proteins in both BT-549 and MCF-7 breast cancer cell lines.The results (Figure 4) demonstrate a decrease in both the phosphorylation levels and the total protein levels.

For BT-549 cells, the decrease in the MAPK/JNK proteins are as follows: pTak1 (16%, *p* < 0.005), Tak1 (34%, *p* < 0.05), p-MKK7 (20%, *p* < 0.005), MKK7 (29%, *p* < 0.001), *p*-JNK (24%, *p* < 0.05), JNK (35%, *p* < 0.005), p-c-Jun (37%, *p* < 0.05), c-Jun (38%, *p* < 0.001), and TNF-α (35%, *p* < 0.05).

For MCF-7 cells, the decrease in the MAPK/JNK proteins are as follows: pTak1 (6%), Tak1 (16%, *p* < 0.05), p-MKK7 (26%, *p* < 0.005), MKK7 (25%, *p* < 0.001), p-JNK (9%, *p* < 0.005), JNK (16%, *p* < 0.001), p-c-Jun (18%, *p* < 0.005), c-Jun (19%, *p* < 0.05), and for TNF-α (1.0%).

### 2.5. Knockdown of PKC-ι Using siRNA Inhibits the MAPK/JNK Pathway Proteins

**Figure 5:**
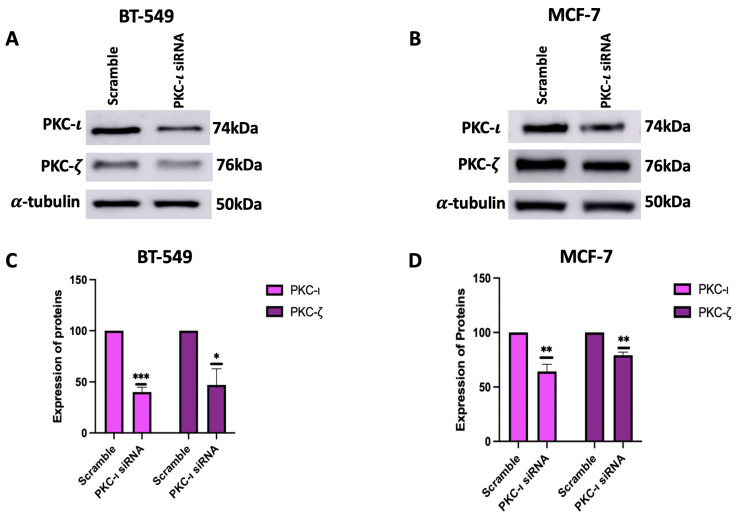
(**A**,**B**) Western blot images and (**C**,**D**) corresponding graphical representation demonstrating effect of PKC-ι knockdown on aPKCs in BT-549 and MCF-7 cells. Treatment with PKC-ι siRNA lowered the levels of PKC-ι in both cell lines, as well as lowered the levels of PKC-ζ in both cell lines. Expression levels were normalized to the corresponding loading controls such as α-tubulin. The graph represents the Mean ± Standard Error of Mean (SEM) of 3 independent experiments of each cell line. *** *p* < 0.001; ** *p* < 0.005, * *p* < 0.05.

To further confirm the effects of PKC-ι on the regulation of MAPK/JNK pathway, PKC-ι was silenced using small interfering RNA (siRNA) specific to PKC-ι. Initially, the efficiency of siRNA mediated knockdown was evaluated by analyzing the expression levels of aPKCs, with an emphasis on the extent of PKC-ι suppression and its possible impact on the expression levels of PKC-ι. The results (Figure 5) indicate that treatment with siRNA significantly reduced both the levels of PKC-ι (60%, *p* < 0.001) and PKC-ζ (53%, *p* < 0.05) for BT549 cells. Similarly, for MCF-7 cells, a reduction in both PKC-ι (35%, *p* < 0.005) and PKC-ζ (21%, *p* < 0.005) was observed.

**Figure 6:**
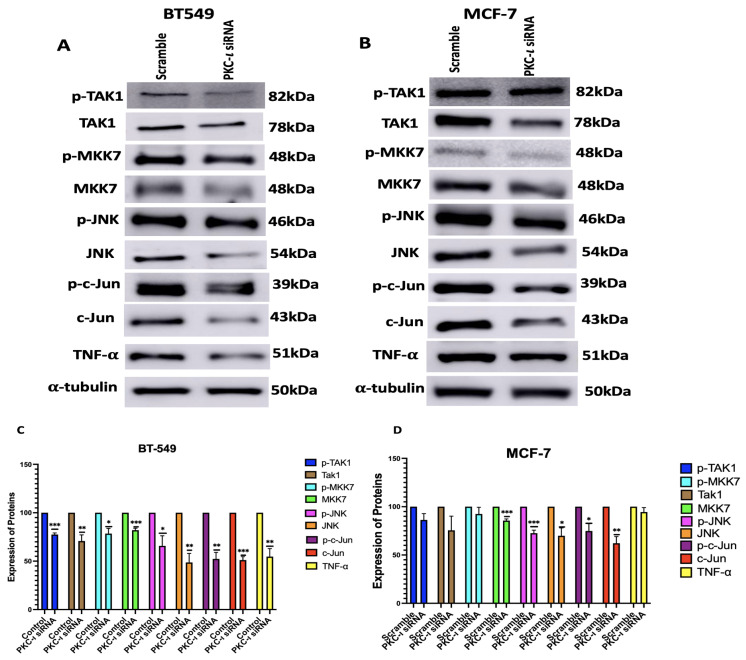
(**A**,**B**) Western blot images and (**C**,**D**) corresponding graphical representation demonstrating the effect PKC-ι knockdown on the expression of MAPK-JNK pathway proteins and their phosphorylated form in BT-549 and MCF-7 cells. Expression levels were normalized to the corresponding loading controls such as β-actin and α-tubulin. Graphs represents the Mean ± Standard Error of Mean (SEM) of 3 independent experiments of each cell line. *** *p* < 0.001; ** *p* < 0.005, * *p* < 0.05.

Subsequently, the effect of PKC-ι knockdown by siRNA was observed in the expression levels of MAPK/JNK pathway proteins. The results (Figure 6) demonstrated a reduction in both the phosphorylation levels and the total protein levels of the MAPK/JNK pathway proteins because of PKC-ι knockdown. For BT-549 cells, the decrease in the MAPK/JNK proteins are as follows: pTak1 (23%, *p* < 0.001), Tak1 (29%, *p* < 0.005), p-MKK7 (22%, *p* < 0.05), MKK7 (18%, *p* < 0.001), p-JNK (34%, *p* < 0.05), JNK (51%, *p* < 0.005), p-c-Jun (47%, *p* < 0.005), c-Jun (48%, *p* < 0.001) and TNF-α (45%, *p* < 0.005). For MCF-7 cells, the decrease in the MAPK/JNK proteins are as follows: pTak1 (14%), Tak1 (25%), p-MKK7 (8%), MKK7 (14%, *p* < 0.001), p-JNK (27%, *p* < 0.001), JNK (30%, *p* < 0.05), p-c-Jun (25%, *p* < 0.05), c-Jun (38%, *p* < 0.005), and for TNF-α (6%).

### 2.6. Co-Localization and Strong Association Was Established Between PKC-ι and c-Jun

**Figure 7:**
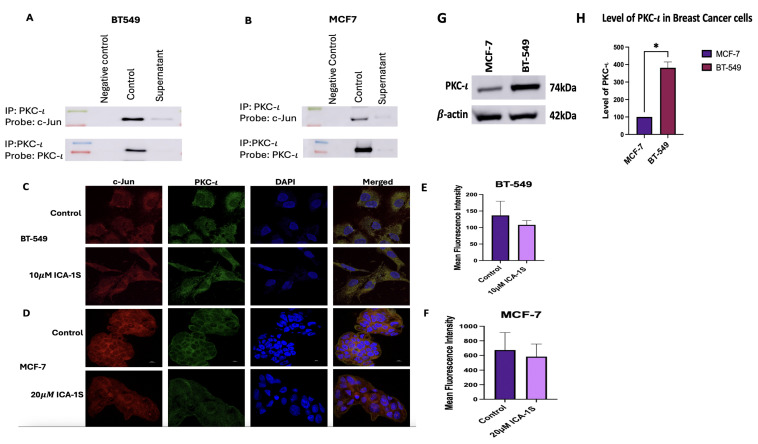
(**A**,**B**) Co-immunoprecipitation of c-Jun with PKC-ι in BT-549 and MCF-7 cell line. Representative Western blot images indicate that there is a direct association between PKC-ι and c-Jun for both the cell lines. (**C**–**F**) Immunofluorescence staining and visualization at 100X magnification of c-Jun protein with 4’,6-diamidino-2-phenylindole (DAPI) staining in BT-549 and MCF-7 cell lines. Treatment with ICA-1S also reduces the mean fluorescence intensity of c-Jun when compared to the control. (**G**,**H**) Level of PKC-ι in MCF-7 and BT-549 cells. MCF-7 cells demonstrate lower levels of PKC-ι, compared to BT-549 cells. The graph represents the Mean ± Standard Error of Mean (SEM) of 3 independent experiments of each cell line. * *p* < 0.05.

To determine if there is any direct association between PKC-ι and c-Jun, PKC-ι was immunoprecipitated and probed with c-Jun. The results (Figure 7A,B) indicate that there is a strong association between PKC-ι and c-Jun for both BT-549 and MCF-7 breast cancer cell lines. In addition, immunofluorescence staining and visualization results (Figure 7C–F) demonstrate the co-localization of PKC-ι and c-Jun. Furthermore, the results also indicate a reduction in the mean fluorescent intensity with ICA-1S treatment.

### 2.7. Baseline Levels of PKC-ι Varies Between Breast Cancer Cell Lines

Baseline levels of PKC-ι were analyzed using Western blotting and the results indicate significantly lower levels of PKC-ι in MCF-7 cells, when compared to the BT-549 cells as shown in (Figure 7G,H). BT-549 cells demonstrate approximately 380% (*p* < 0.05) increase in the levels of PKC-ι, when compared to MCF-7 cells.

### 2.8. ICA-1S May Accelerate the Degradation of c-Jun

**Figure 8:**
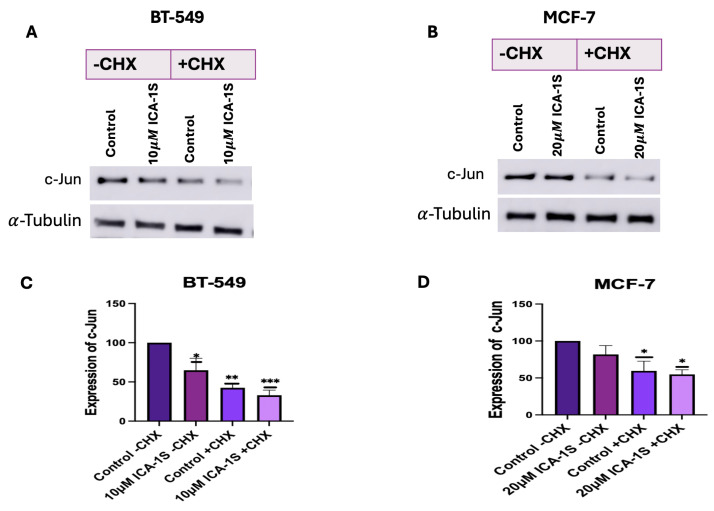
(**A**,**B**) Western blot images and (**C**,**D**) corresponding graphical representation demonstrating the expression of c-Jun protein with and without CHX treatment for BT-549 and MCF-7 cells. An enhanced decrease in the levels of c-Jun is observed after CHX treatment in the ICA-1S treatment group. Expression levels are normalized to the corresponding loading control such as α-tubulin. Graphs represents the Mean ± Standard Error of Mean (SEM) of 3 independent experiments of each cell line. *** *p* < 0.001; ** *p* < 0.005, * *p* < 0.05.

The CHX chase assay was conducted to determine whether ICA-1S promotes the degradation of c-Jun. A decrease in c-Jun expression levels in the ICA-1S treatment group after cycloheximide treatment indicates that ICA-1S might facilitate the degradation of c-Jun. The results (Figure 8) demonstrate a 10% decrease in BT-549 cells and 5% decrease in MCF-7 cells in the expression levels of c-Jun in the ICA-1S treated group after four hours post cycloheximide treatment when compared to the cycloheximide-treated control group.

### 2.9. Reverse Transcription Quantitative PCR (RT-qPCR)

**Figure 9:**
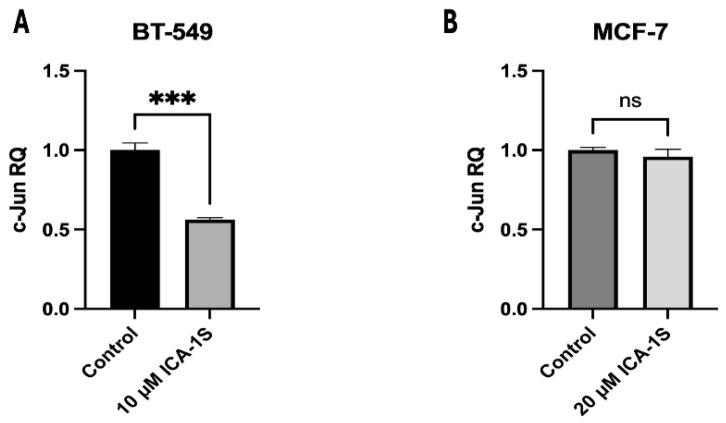
Expression of c-Jun mRNA in (**A**) BT-549 and (**B**) MCF-7 cell line. The graph representing BT-549 cells demonstrate the relative quantification value (RQ) of the target gene (c-Jun) is 0.5, which indicates a twofold downregulation in comparison to the control group, following normalization with the reference gene, GAPDH. However, no significant downregulation was observed for MCF-7 cells. Graphs represent the Mean ± Standard Error of Mean (SEM) of 3 independent experiments of each cell line. *** *p* < 0.001, ns: not significant.

RT-qPCR was conducted was to determine the effect of ICA-1S in the c-Jun mRNA levels for both BT-549 and MCF-7 breast cancer cells. The results (Figure 9) indicate 50% reduction in the c-Jun mRNA levels in the ICA-1S treated group when compared to the control. However, for MCF-7 cells, no significant changes were observed.

## 3. Discussion

The overexpression of PKC-ι has been previously implicated in regulating various types of cancer cell proliferation, survival, migration and invasion, including ovarian, melanoma, and colon cancer [2,14,15,16]. Among the various signaling pathways that control these cellular processes, the MAPK/JNK pathway plays a particularly substantial oncogenic role. The MAPK/JNK pathway has been previously established to play a role in the metastasis and tumorigenesis of different types of cancer, including breast cancer [17]. A key downstream effector of the MAPK/JNK pathway is the transcription factor c-Jun, which is phosphorylated and activated via this signaling cascade. Activated c-Jun, which is an essential component of the AP-1 transcription complex, plays a key role in regulating proteins that are crucial for cancer cell survival and proliferation [18].

This project demonstrates the role of PKC-ι in regulating the oncogenic MAPK/JNK pathway, thereby activating the c-Jun and AP-1 transcription complex, which further governs the TNF-α mediated activation of the MAPK/JNK.

ICA-1S is a PKC-ι specific allosteric inhibitor, which is a nucleoside analog of ICA-1T. Previous studies have demonstrated that ICA-1T binds to amino acid residues 469-475 of PKC-ι, thereby inhibiting the function of PKC-ι [19]. Previous studies by the Acevedo-Duncan group also demonstrated the molecular docking of both ICA-1T and ICA-1S to structures of PKC-ι and identified an allosteric binding pocket [14]. The pocket was located within the C-lobe of the kinase domain and was framed by solvent-exposed residues of helices αF-αI and the activation segment [14]. While ICA-1S has been observed to inhibit the activity of PKC-ι in several types of cancer, including ovarian and breast cancer, the exact binding site is yet to be established. Current studies conducted by the Acevedo-Duncan group are based on the specific interaction of ICA-1S and PKC-ι. The binding events of ICA-1S and PKC-ι is demonstrated as a possible entropy-driven spontaneous hydrophobic interaction, which is validated by thermodynamics [20]. In addition, this study also established the possibility of stronger binding of ICA-1S with PKC-ι, compared to ICA-1T [20]. The Acevedo-Duncan group also previously reported preclinical testing of chronic ICA-1S exposure, where the stability and toxicity of ICA-1S were evaluated. The results concluded that ICA-1S is stable and has low toxicity [21]. In this study, we examine the effect of ICA-1S on breast cancer cell lines. Here, we demonstrate that the inhibition of PKC-ι by ICA-1S reduces the cell proliferation of BT-549 (Triple Negative subtype) and MCF-7 (Luminal A subtype) breast cancer cells. The concentration at which 50% inhibition (IC_50_) was observed (Figure 1) was 10μM and 20μM for BT-549 and MCF-7 cells, respectively. Although the concentration range used is consistent with previous reports, the possibility of off-target effects at higher doses remains, and future studies that include kinase activity assays or kinase profiling will help validate the target specificity more conclusively.

Nevertheless, consistent with the cell proliferation findings, it was also demonstrated that ICA-1S induced apoptosis in breast cancer cell lines as shown by the cleavage of Caspase 3 and PARP. The results indicate that treatment with ICA-1S has increased the expression levels of cleaved Caspase 3 and cleaved PARP (Figure 2A,B) in breast cancer cells. Cleavage of Caspase 3 and PARP are hallmark events in apoptosis, indicating the activation of apoptotic pathways [22]. The expression of cleaved PARP could only be observed for the MCF-7 cells, as these cells lack functional Caspase 3 expression. This deficiency is caused by a deletion within the CASP3 gene, resulting in the skipping of exon 3 and the introduction of a premature stop codon, which prevents the translation of a functional Caspase 3 protein [23]. Although, MCF-7 lacks the Caspase 3 protein, it still can undergo apoptosis as previously reported. One way that MCF-7 cells undergo apoptosis is via the intrinsic apoptotic pathway, which involves the regulation of the apoptotic cascades by changes in the mitochondria, resulting in the release of cytochrome C into the cytosol and the activation of the initiator caspase 9, which then further activates caspase 7 [24]. Caspase 7 can cleave PARP in the absence of caspase 3 [25]. In our study we observed increased levels of cleaved PARP in MCF-7 cells with ICA-1S treatment, suggesting the activation of such a caspase-3-independent apoptotic pathway, which is consistent with the known mechanism in which caspase 9 can cleave caspase 7, which can in turn cleave PARP. In addition, as observed in (Figure 2B,D), the levels of PARP remained unchanged for the MCF-7 cells, even when the levels of cleaved PARP increased. This could be due to the cleaved fragments not undergoing substantial degradation, or new PARP protein synthesis could be compensating for the degraded protein, resulting in increased levels of cleaved PARP without a reduction in the total PARP levels. This can also be cell-line specific, as various cell lines exhibit different reactions to apoptotic stimuli, stages of cell cycle, and other cellular conditions that can affect how PARP levels fluctuate. Furthermore, flow cytometry was also conducted to confirm apoptosis (Figure 2E–H), which further confirmed the induction of apoptosis by ICA-1S treatment, demonstrated by the increase in the percentage of early and late apoptosis in the treated samples.

To further assess the effect of ICA-1S on the MAPK/JNK signaling pathway, initially, the levels of aPKCs were analyzed after treatment with ICA-1S. The results (Figure 3A–D) indicated a significant reduction in the levels of PKC-ι for both cell lines after treatment with ICA-1S. On the contrary, the levels of PKC-ζ decreased for BT-549 cell lines, but there was no significant change in the PKC-ζ levels for MCF-7 cells. The differences in PKC-ζ expression can also be due to the differences in the levels of the aPKC isoforms in the cell line. BT-549 cell lines have a higher level of both aPKC isoforms when compared to the MCF-7 cells. The inhibition of PKC-ι by ICA-1S may destabilize PKC-ζ, as it might be an interacting or co-regulated partner of PKC-ι, which can lead to co-degradation or the compensatory downregulation of PKC-ζ. Although ICA-1S is a PKC-ι-specific inhibitor, the levels of PKC-ζ are seen to be affected in BT-549, which may be because PKC-ι regulates the expression of PKC-ζ. Studies have previously established that the functional PKC module consists of inter-PKC regulations via feedback loops and sequential activation [26]. This suggests the possibility of different PKC isoforms regulating one another and indicates that changes in one isoform’s expression level can affect the other’s expression level as well. On the contrary, MCF-7 cells have a lower expression of PKC-ι and relies less on PKC-ζ driven survival pathways. In addition, since BT549 cells are more aggressive and belong to the triple negative subtype, they might rely on PKC-ι signaling more, and therefore these signaling networks might be more tightly regulated by stress-response pathways that respond to PKC-ι inhibition by downregulating the affected isoform to limit irregular signaling. MCF-7 cells are hormone dependent and less aggressive and may have a more robust feedback mechanism that stabilizes or increases the levels of PKC-ζ to maintain cell polarity and survival when PKC-ι is inhibited.

Subsequently, we examined the effect of PKC-ι inhibition by ICA-1S on the MAPK/JNK pathway proteins (Figure 4), and we demonstrated that there are decreased expression levels for all the MAPK/JNK pathway proteins, c-Jun; along with that, we observed a reduction in the expression levels of TNF-α.

As previously mentioned, the transcription factor c-Jun is an integral member of the AP-1 complex [6]. AP-1 activity is correlated with a steady increase in the c-Jun expression levels. c-Jun promoter contains high affinity AP-1 binding site [6]. A regulatory circuit exists between c-Jun and AP-1, where AP-1 can activate the c-Jun promoter and gene expression; in turn, c-Jun expression can further enhance AP-1, which further potentiates its own gene promoter expression [6,27]. Therefore, this positive feedback loop enables c-Jun to prolong and amplify AP-1 activity [6,27]. In addition, the JUN family predominates in the AP-1 complex; therefore, it can be stated that a reduction in c-Jun expression also leads to the downregulation of AP-1 activity [28]. c-Jun/AP-1 is a transcription factor for TNF-α. Studies have previously shown that c-Jun regulates nearly a third of the TNF-α-regulated transcriptome [13]. They also discovered that there were 13,800 binding regions in the cistrome for the AP-1 transcription factor c-Jun in the TNF-α-stimulated TNBC cells and that AP-1 plays a crucial role in the TNF-α-mediated TNBC progression [13]. This was also substantiated by our results, which indicate that the reduction in c-Jun and AP-1 expression levels also leads to the downregulation of TNF-α, with a more prominent effect observed in the BT-549 cells, which are TNBC cells (Figure 4).

TNF-α further activates transforming growth factor-activated kinase-1 (TAK1), which is a family of mitogen-activated protein kinase kinase Kknase (MAP3K). The binding of TNF-α to its receptor Tumor Necrosis Factor Receptor 1 (TNFR1) recruits TNFR associated protein with a death domain (TRADD), which forms a receptor complex by recruiting TNF receptor associated factor 2 and 5 (TRAF2 and TRAF5), cellular inhibitor of apoptosis 1 and 2 (CIAP1 and CIAP2), and receptor-interacting protein 1 (RIP1). TRAF2 and TRAF5 further catalyze the k63-linked polyubiquitination of RIP1 [29]. The k63-linked polyubiquitination chain recruits TAK1 binding protein 2 and 3 (TAB 2 and TAB3), which then forms a signal complex with TAK1 and activates TAK1 [29]. TAK-1 then phosphorylates and activates mitogen-activated protein kinase kinase 7 (MKK7), which belongs to the mitogen-activated protein kinase kinase (MAP2K) family, which is followed by the phosphorylation and activation of JNK, a member of the MAPK family, by MKK7. Subsequently, JNK activates c-Jun through phosphorylation [29,30]. The inhibition of PKC-ι by ICA-1S has inhibited each of these proteins in the oncogenic positive feedback loop as demonstrated in our results (Figure 4), indicating that PKC-ι regulates this entire signaling cascade.

To further validate the results with ICA-1S and to ensure that the observed downregulation of MAPK/JNK protein expressions was due to the inhibition of PKC-ι, we inhibited PKC-ι using siRNA. Our results (Figure 5) indicate that treatment with PKC-ι specific siRNA also inhibited PKC-ζ in both breast cancer cell lines, which supports the theory that PKC-ι plays a role in regulating PKC-ζ. When treated with ICA-1S, the MCF-7 cells did not demonstrate any significant change in PKC-ζ (Figure 3B,D); however, when treated with the PKC-ι specific siRNA, the levels of PKC-ζ decreased. This might be because ICA-1S and siRNA have different modes of action. ICA-1S binds to the catalytic domain of PKC-ι and inhibits the functional activity of PKC-ι and siRNA completely inhibits PKC-ι protein translation, which leads to the gene silencing of PKC-ι. As previously mentioned, MCF-7 cells express low levels of PKC-ι, making them less sensitive to ICA-1S treatment. The reduced dependence on PKC-ι signaling also correlates to less dependence on PKC-ζ-driven survival pathways. However, since siRNA completely knocks down the PKC-ι gene, it not only inhibits its catalytic function but also inhibits its stabilizing roles within signaling complexes. Consequently, the complete knockdown of PKC-ι might cause the co-degradation or inhibition of PKC-ζ.

We also observed that the knockdown of PKC-ι effectively reduces the expression levels of MAPK/JNK pathway proteins (Figure 6). Moreover, consistent with the findings presented in Figure 4, the siRNA-mediated knockdown of PKC-ι further demonstrates a more prominent decrease in the expression levels of the MAPK/JNK proteins in the BT-549 cells, compared to the MCF-7 cells, which can be attributed to the lower levels of PKC-ι in the MCF-7 cells as previously mentioned. This further affirms that PKC-ι regulates this entire signaling cascade. It was also observed that the decrease in the phosphorylated forms of the MAPK/JNK proteins are not as significant as the decrease in the total protein levels. This might be because the total protein levels and phosphorylation status might be regulated by distinct mechanism. The phosphorylation status of a protein can be independently regulated within a cellular signaling pathway, whereas changes in the total protein expression levels might occur in response to signals or stress. In addition, phosphorylated proteins are either selectively protected from degradation or stabilized by feedback mechanisms in certain signaling networks.

To further substantiate the proposed hypothesis, we examined whether PKC-ι is associated with c-Jun, via which PKC-ι might regulate this signaling cascade. Our results demonstrated that PKC-ι is strongly associated with c-Jun (Figure 7A,B) in both breast cancer cell lines. In addition, we demonstrated co-localization of PKC-ι and c-Jun by immunofluorescence staining and examined the reduction in mean fluorescence intensity after treatment with ICA-1S (Figure 7C–F). These results suggest that PKC-ι is regulating the signaling cascade via interaction with c-Jun. Furthermore, the results demonstrated in (Figure 7G,H) further substantiates the results found in (Figure 4, where we observed the decrease in the levels of MAPK/JNK proteins was more prevalent in the BT-549 cell lines compared to the MCF-7 cells.This could be due to the low levels of PKC-ι in the MCF-7 cells, which was further validated by Western blot analysis by comparing the baseline levels of PKC-ι in both MCF-7 and BT-549 as shown in (Figure 7G,H). Since ICA-1S is a PKC-ι specific inhibitor, lower levels of PKC-ι would lead to a reduced effect of ICA-1S. Therefore, the effect of ICA-1S is less in MCF-7 cells compared to BT-549 cells, which can be attributed to the varying levels of PKC-ι in each cell line.

We also examined whether ICA-1S can facilitate the degradation of c-Jun by using the CHX chase assay. CHX primarily inhibits protein synthesis by interrupting the translocation step during translation elongation. It disrupts the movement of peptidyl-tRNA on the mRNA-ribosomal complex and prevents the elongation of the chain [31]. Our results (Figure 8A–D) indicate a decrease in the c-Jun level in the ICA-1S-treated group after CHX treatment. Once CHX blocks protein synthesis, degradation by the proteasome or lysosome reduces the amount of intracellular protein [31]. Therefore, in the absence of protein synthesis, any further reduction in c-Jun expression in the ICA-1S treatment group compared to the control group is an indication that ICA-1S might be promoting the degradation of c-Jun.

Lastly, we examined whether ICA-1S affects c-Jun at the transcriptional level by measuring c-Jun mRNA levels in the ICA-1S-treated group, in comparison to the control group. Our results (Figure 9A,B) indicate that treatment with ICA-1S reduces the level of c-Jun mRNA in BT-549 cell line; however, it demonstrates no significant change in the mRNA levels of MCF-7 cells. These findings are consistent with our observations in (Figure 4), where a less pronounced reduction was observed with ICA-1S in the MAPK/JNK proteins in MCF-7 cells compared to the BT-549 cells. This may also be a consequence of the varying levels of PKC-ι expression across different cell lines, which could affect the extent to which ICA-1S exerts its effects, given that ICA-1S is a PKC-ι specific inhibitor as previously mentioned. This also supports our theory that varying levels of PKC-ι might be the reason why ICA-1S has less effect on MCF-7 breast cancer cells. Moreover, MCF-7 is a luminal A breast cancer subtype, which means it is a hormone-dependent cell line (estrogen and progesterone positive). Therefore, MCF-7 cell progression and survival may be regulated by hormones more than they are by PKC-ι.

On the contrary, BT-549 cell line is a TNBC cell line and is negative for estrogen, progesterone, and human epidermal growth factor (HER2) negative. Previous studies have demonstrated that PKC-ι signaling is highly active during breast cancer invasive progression and metastatic breast cancers, which are the advanced stages of breast cancer that are more frequently observed in TNBC patients [32]. Since TNBCs are not hormone dependent, they can be significantly regulated by PKC-ι signaling, which might be the reason why the inhibition of PKC-ι has a greater effect on the BT-549 cell line.

## 4. Materials and Methods

### 4.1. Reagents, Antibodies, and Inhibitors

5-amino-1-(1R, 2S, 3S, 4R)-2,3-dihydroxy-4-methylcyclopentyl)-1H-imidazole-4- carboxamide (ICA-1S) was obtained from DC Chemicals (Shanghai, China). Primary antibodies were obtained as follows: Antibodies against PKC-ι (610175, BD Biosciences, Franklin Lakes, NJ, USA), PKC-ζ (sc-17781, Santa Cruz Biotechnology, Dallas, TX, USA), Cleaved Caspase-3 (Asp175; 9661; Cell Signaling Technology, Danvers, MA, USA), Caspase-3 (9662; Cell Signaling Technology), Cleaved PARP (Asp214; 9541; Cell Signaling Technology), PARP (9542; Cell Signaling Technology), c-Jun (9165S; cell signaling technology), p-c-Jun (S73; 3270S; cell signaling technology), phospho-JNK (sc-6254; Santa Cruz Biotechnology) JNK (sc-7345; Santa Cruz Biotechnology), phospho-Tak1 (Thr 187; cell signaling technology #4536), Tak1 (4505; cell signaling technology), phosphor-MKK7 (Ser271/Thr275; 4171; cell signaling technology), MKK7 (4172; Cell Signaling Technology), TNF-α (3707; Cell Signaling Technology),β-actin (A3854, Sigma-Aldrich; Merck KGaA, St. Louis, MO, USA), α-Tubulin (#3873, Cell Signaling Technology).

PKC-ι agarose conjugated beads (sc-17837 AC; Santa Cruz Biotechnology) was used for co-immunoprecipitation. c-Jun antibody (sc-166540 AF546) conjugated with Alexa Fluor 546 and PKC-ι antibody (sc-17837 AF488) conjugated with Alexa Fluor 488 for immunofluorescence.

PKC-ι (PRKCI) Human siRNA Oligo Duplex (SR321426, Origene, Rockville, MD, USA), siTran 2.0 siRNA transfection reagent (TT320002, Origene) was used for siRNA treatments to knockdown PKC-ι. Trilencer-27 universal scrambled siRNA Duplex (SR30004, Origene) was used as a negative control.

### 4.2. Breast Cancer Cell Lines

BT-549 (ATCC® HTB-122™) and MCF-7 (ATCC® HTB-22™) breast cancer cell lines were purchased from the American Type Tissue Culture Collection (ATCC; Manassas, VA, USA). Once the cell lines were received, the first few passages were cryo-preserved in liquid nitrogen, and these cells were later used for cell culture. Cells were cultured at 37 °C and 5% carbon dioxide (CO_2_). The BT-549 cells were cultured in Roswell Park Memorial Institute-1640 (RPMI-1640) base medium (A1049101, Gibco™, Waltham, MA, USA), and the MCF-7 cells were cultured in Eagle’s Minimum Essential Medium (MEM) (10-009-CV, Corning, NY, USA). Both RPMI-1640 and MEM were supplemented with 10% fetal bovine serum (FBS), 1% penicillin (10,000 IU/mL) and streptomycin (10,000 μg/mL) antibiotics (Corning, AZ, USA) and insulin, human recombinant, zinc solution (Gibco™, Waltham, MA, USA). Both the cell lines were seeded and grown as monolayers in T75 flasks. Once 70–80% confluent, the cells were lifted using trypsin-Ethylenediaminetetraacetic acid (EDTA) and neutralized using equal volumes of base medium and then used for experiments.

### 4.3. Cell Proliferation Assay

Both the BT-549 and MCF-7 cell lines were cultured in T25 cell culture flasks with, approximately 80,000 cells seeded into each. The flasks were treated with various concentrations of ICA-1S (2.5 μM, 5 μM, 10 μM, 20 μM, 30 μM), in addition to an untreated control set. The treatment was repeated over a period of 120 h (5 days), and cells were treated at 24 h intervals. Consequently, live cells were counted using the Nexcelom Bioscience Cellometer Auto T4 Plus Cell Counter (Revvity, MA, USA). The dosage of ICA-1S at which there was a 50% reduction in cell proliferation of the breast cancer cells was identified as IC_50_ value.

### 4.4. Sample Processing, Protein Quantification and Western Blotting

Cells were seeded in 100 mm plates and grown to 30–40% confluency. The cells were then treated with the IC_50_ concentration of ICA-1S for 5 days, followed by the collection of cell lysate using lysis buffer (C7027, Invitrogen, Waltham, MA, USA). Subsequently, the cells were processed by sonication and centrifugation (at 12,000 rpm, for 20 min at 4 °C), and a Bradford assay was conducted for protein quantification. For Western blot analysis, equal amounts of (80–100 μg) protein from cell lysates were loaded in polyacrylamide gels and separated by sodium dodecyl sulfate-polyacrylamide gel electrophoresis (SDS-PAGE). The next step was to transblot the proteins onto a 0.2 μm nitrocellulose membrane, followed by incubation of the membrane in a 4% milk-blocking solution [33]. The membranes were incubated overnight with the primary antibodies, followed by the addition and incubation with the secondary antibodies. Band intensity was quantified using the Amersham Imager 600 software (GE Healthcare, Chalfont St. Giles, UK) with automatic exposure settings. Expression levels were normalized to the corresponding loading controls, such as β-actin and α-tubulin.

### 4.5. Determining Baseline Levels of PKC-ι

Both cell lines were cultured until 80–90% confluency and collected using lysis buffer. The lysates were then processed, and Bradford assay was conducted for protein quantification. The levels of PKC-ι were then analyzed using SDS-PAGE and Western blotting.

### 4.6. Small Interfering RNA (siRNA) Treatment

BT-549 and MCF-7 cell lines were cultured in 100 mm plates and grown to 60–70% confluency. Subsequently, the cells were treated with 100 nM of either PKC-ι-specific siRNA or scrambled siRNA and incubated for an additional 48 h. SDS-PAGE and Western blotting were then performed to investigate the effect of PKC-ι knockdown on the MAPK/JNK signaling pathway.

### 4.7. Co-Immunoprecipitation (IP)

Cell lysates were collected and processed. The protein concentration was quantified using a Bradford assay. PKC-ι was immunoprecipitated from 1000 μg of protein samples of different cell lines with PKC-ι-specific antibody conjugated with agarose. After the addition of PKC-ι-specific beads to the cell lysate and overnight incubation, the lysates were washed to remove unbound proteins, the following day. The first supernatant was collected to assess the level of dissociated protein. After three washes, the protein complex was then eluted from the beads, and the interaction between proteins was analyzed using SDS-PAGE and Western blotting.

### 4.8. Immunofluorescence

Approximately 3000 cells were seeded in 4-chamber slides (229164, CELLTREAT, Ayer, MA, USA) and were treated with IC_50_ concentration of ICA-1S. The cells were fixed with 4% paraformaldehyde and permeabilized with 0.1% Triton X-100 following a 5-day ICA-1S treatment. The cells were then blocked with 5% BSA (bovine serum albumin), followed by the addition and incubation with Alexa Fluor 546-conjugated c-Jun antibody (red) and Alexa Fluor 488-conjugated PKC-ι (green) antibody. Subsequently, cells were washed and mounted with DAPI (4’,6-diamidino-2-phenylindole). Fluorescent signals were detected with a Nikon confocal fluorescence microscope (Melville, NY, USA). NIS-Elements imaging software version 5.21.00 (Nikon Instruments Inc., Melville, NY, USA) was used to acquire all the images, and ImageJ software 1.54 g was used for analysis [34]. The fluorescence level was measured using the following formula: the mean fluorescence intensity (MFI) is equal to the MFI of an area of interest (ROI) minus the MFI of the background [35].

### 4.9. Flow Cytometry

The cells were harvested after treatment with ICA-1S. Cells were washed and resuspended in 1X binding buffer, which was followed by the addition and incubation of 5 μL fluorochrome-conjugated Annexin V to 100 μL of the cell suspension for 10–15 min. Subsequently, 2 mL of 1X binding buffer was added, and the suspension was centrifuged at 400–600 rpm for 5 min, and the supernatant was discarded. Cells were again resuspended in 200 μL of 1X binding buffer, then 5 μL of propidium iodide staining solution was added, and the samples were incubated for 5–15 min on ice. Cells were then analyzed by flow cytometry using a BD LSR II cytometer (BD Biosciences, Franklin Lakes, NJ, USA).

### 4.10. Cycloheximide Chase Assay

Cells were seeded in 100 mm plates and treated for 5 days with IC_50_ concentration of ICA-1S for both cell lines. On the day of collection, cells were treated with 50 μg of CHX solution (Sigma-Aldrich, St. Louis, MO, USA), and cells were collected after 4 h and processed. SDS-PAGE and Western blotting were then conducted, and the effect of CHX treatment was analyzed.

### 4.11. Reverse Transcription Quantitative PCR (RT-qPCR)

Total RNA was isolated from BT-549 and MCF-7 cell lines using RNAzol RT (Sigma-Aldrich, Cat# R4533, St. Louis, MO, USA) according to the manufacturer’s instructions. RNA quality was assessed using 260/230 and 260/280 absorbance ratios to ensure purity. A total of 1 μg of RNA was reverse-transcribed into cDNA using the iScript™ cDNA Synthesis Kit according to the manufacturer’s protocol (Bio-Rad, Hercules, CA, USA; Catalog #1708891). RT-qPCR was performed using a Power SYBR Green PCR Master Mix (Thermo Fisher Scientific, Waltham, MA, USA; Catalog #4367659) and run on a sequence detection system (Applied Biosystems, Foster City, CA, USA). RT-qPCR reactions were performed in triplicate. Primer concentrations were optimized for a single melt curve and consistent amplification. The following primer sequences were used: c-Jun (F: 5’-CCTTGAAAGCTCAGAACTCGGAG-3’; R: 5’-TGCTGCGTTAGCATGAGTTGGC-3’) and GAPDH (F: 5’-GATCATCAGCAATGCCTCCT-3’; R: 5’-TGTGGTCATGAGTCCTTCCA-3’). Relative transcript expression levels were determined using the ▵▵CT method, normalizing target gene expression to GAPDH.

### 4.12. Statistical Analysis

The statistical analysis was carried out with GraphPad Prism version 10.3.1. For comparisons involving only two groups, an unpaired two-tailed *t*-test was applied to assess statistical significance. Data are presented as bar graphs showing the mean ± standard error of the mean (SEM) from three independent experimental replicates. A one-way ANOVA test was used to evaluate the differences between various treatment groups and the control.

## 5. Conclusions

**Figure 10:**
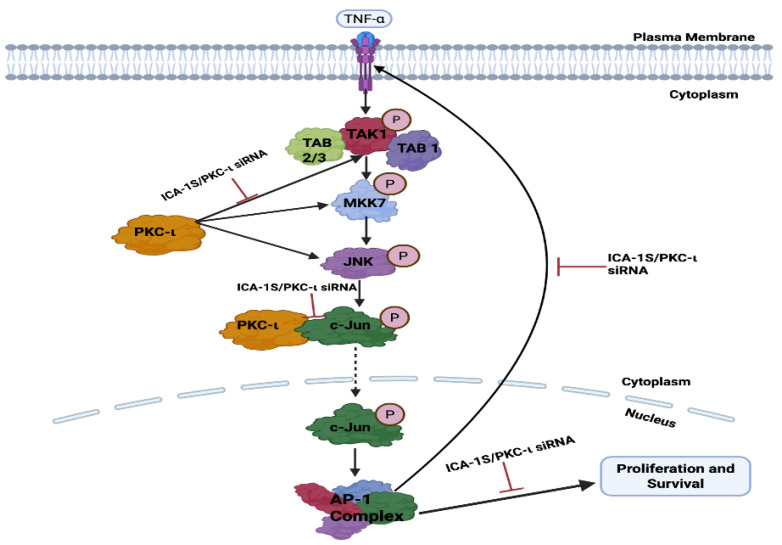
Schematic representation of the possible pathway via which PKC-ι regulates MAPK/JNK pathway proteins in breast cancer. The inhibition of PKC-ι leads to c-Jun degradation or reduced expression, which decreases the AP-1 activity. Decrease in AP-1 activity reduces the levels of TNF-α, TAK-1, MKK7 and JNK. Decrease in the levels of JNK activity further decreases c-Jun expression, thereby reinforcing the oncogenic positive feedback loop. All these proteins play an oncogenic role in breast cancer proliferation and survival; therefore, reducing the levels of these proteins will lead to decreased proliferation and survival.

PKC-ι plays a crucial role in regulating the oncogenic MAPK/JNK loop. The inhibition of PKC-ι by ICA-1S reduces breast cancer cell proliferation and induces apoptosis as observed in (Figure 10), highlighting PKC-ι as a potential therapeutic target.

## Data Availability

Data are contained within the article.

## References

[1] World Health Organization (2024). Breast Cancer. https://www.who.int/news-room/fact-sheets/detail/breast-cancer.

[2] Garg R., Benedetti L.G., Abera M.B., Wang H., Abba M., Kazanietz M.G. (2014). Protein kinase C and cancer: What we know and what we do not. Oncogene.

[3] Marengo B., De Ciucis C.G., Ricciarelli R., Pronzato M.A., Marinari U.M., Domenicotti C. (2011). Protein kinase C: An attractive target for cancer therapy. Cancers.

[4] Webb B.L., Hirst S.J., Giembycz M.A. (2000). Protein kinase C isoenzymes: A review of their structure, regulation and role in regulating airways smooth muscle tone and mitogenesis. Br. J. Pharmacol..

[5] Kojima Y., Akimoto K., Nagashima Y., Ishiguro H., Shirai S., Chishima T., Ichikawa Y., Ishikawa T., Sasaki T., Kubota Y. (2008). The overexpression and altered localization of the atypical protein kinase C lambda/iota in breast cancer correlates with the pathologic type of these tumors. Hum. Pathol..

[6] Meng Q., Xia Y. (2011). c-Jun, at the crossroad of the signaling network. Protein Cell.

[7] Jiao X., Katiyar S., Willmarth N.E., Liu M., Ma X., Flomenberg N., Lisanti M.P., Pestell R.G. (2010). c-Jun induces mammary epithelial cellular invasion and breast cancer stem cell expansion. J. Biol. Chem..

[8] Vleugel M.M., Greijer A.E., Bos R., van der Wall E., van Diest P.J. (2006). c-Jun activation is associated with proliferation and angiogenesis in invasive breast cancer. Hum. Pathol..

[9] Wang J., Kuiatse I., Lee A.V., Pan J., Giuliano A., Cui X. (2010). Sustained c-Jun-NH2-kinase activity promotes epithelial-mesenchymal transition, invasion, and survival of breast cancer cells by regulating ERK activation. Mol. Cancer Res..

[10] Karin M., Liu Z., Zandi E. (1997). AP-1 function and regulation. Curr. Opin. Cell Biol..

[11] Song D., Lian Y., Zhang L. (2023). The potential of activator protein 1 (AP-1) in cancer targeted therapy. Front. Immunol..

[12] Liu Y., Ludes-Meyers J., Zhang Y., Munoz-Medellin D., Kim H.-T., Lu C., Ge G., Schiff R., Hilsenbeck S.G., Osborne C.K. (2002). Inhibition of AP-1 transcription factor causes blockade of multiple signal transduction pathways and inhibits breast cancer growth. Oncogene.

[13] Qiao Y., He H., Jonsson P., Sinha I., Zhao C., Dahlman-Wright K. (2016). AP-1 is a key regulator of proinflammatory cytokine TNF*α*-mediated triple-negative breast cancer progression. J. Biol. Chem..

[14] Ratnayake W.S., Apostolatos C.A., Apostolatos A.H., Schutte R.J., Huynh M.A., Ostrov D.A., Acevedo-Duncan M. (2018). Oncogenic PKC-*ι* activates Vimentin during epithelial-mesenchymal transition in melanoma; a study based on PKC-*ι* and PKC-*ζ* specific inhibitors. Cell Adhes. Migr..

[15] Marzan M., Oishee N.N., Olatunji A.O., Shourav A.H., Noor R.E., Astalos A.J., Leahy J.W., Acevedo-Duncan M. (2025). Proteasome Inhibitor MG-132 and PKC-*ι*-Specific Inhibitor ICA-1S Degrade Mutant p53 and Induce Apoptosis in Ovarian Cancer Cell Lines. Int. J. Mol. Sci..

[16] Khalid K.M., Ratnayake W.S., Apostolatos C.A., Acevedo-Duncan M. (2023). Dual inhibition of atypical PKC signaling and PI3K/Akt signaling dysregulates c-Myc to induce apoptosis in clear cell Renal Cell Carcinoma. Front. Oncol..

[17] Burotto M., Chiou V.L., Lee J., Kohn E.C. (2014). The MAPK pathway across different malignancies: A new perspective. Cancer.

[18] Chen J., Ye C., Wan C., Li G., Peng L., Peng Y., Fang R. (2021). The roles of c-Jun N-terminal kinase (JNK) in infectious diseases. Int. J. Mol. Sci..

[19] Pillai P., Desai S., Patel R., Sajan M., Farese R., Ostrov D., Acevedo-Duncan M. (2011). A novel PKC-iota inhibitor abrogates cell proliferation and induces apoptosis in neuroblastoma. Int. J. Biochem. Cell Biol..

[20] Noor R., Islam S., Smalley T., Keramisanou D., Astalos A.J., Leahy J.W., Gelis I., Acevedo-Duncan M. (2025). Biophysical Insights into the Binding Interactions of Inhibitors (ICA-1S/1T) Targeting Protein Kinase C-*ι*. Preprint.

[21] Apostolatos C.A., Ratnayake W.S., Breedy S., Chuah J.K.C., Miller J.A., Zink D., Bourgeois M., Acevedo-Duncan M. (2024). Preclinical Testing of Chronic ICA-1S Exposure: A Potent Protein Kinase C-*ι* Inhibitor as a Potential Carcinoma Therapeutic. Drugs Drug Candidates.

[22] Elmore S. (2007). Apoptosis: A review of programmed cell death. Toxicol. Pathol..

[23] Tian T. (2023). MCF-7 cells lack the expression of Caspase-3. Int. J. Biol. Macromol..

[24] Jin S., Zhang Q.Y., Kang X.M., Wang J.X., Zhao W.H. (2010). Daidzein induces MCF-7 breast cancer cell apoptosis via the mitochondrial pathway. Ann. Oncol..

[25] Erener S., Pétrilli V., Kassner I., Minotti R., Castillo R., Santoro R., Hassa P.O., Tschopp J., Hottiger M.O. (2012). Inflammasome-activated caspase 7 cleaves PARP1 to enhance the expression of a subset of NF-kappaB target genes. Mol. Cell.

[26] Mukherjee A., Roy S., Saha B., Mukherjee D. (2016). Spatio-Temporal Regulation of PKC Isoforms Imparts Signaling Specificity. Front. Immunol..

[27] Angel P., Hattori K., Smeal T., Karin M. (1988). The jun proto-oncogene is positively autoregulated by its product, Jun/AP-1. Cell.

[28] McCabe L.R., Banerjee C., Kundu R., Harrison R.J., Dobner P.R., Stein J.L., Lian J.B., Stein G.S. (1996). Developmental expression and activities of specific fos and jun proteins are functionally related to osteoblast maturation: Role of Fra-2 and Jun D during differentiation. Endocrinology.

[29] Shi J.H., Sun S.C. (2018). Tumor Necrosis Factor Receptor-Associated Factor Regulation of NF-*κ* B and MAPK Pathways. Front. Immunol..

[30] Morrison D.K. (2012). MAP kinase pathways. Cold Spring Harb. Perspect. Biol..

[31] Miao Y., Du Q., Zhang H., Yuan Y., Zuo Y., Zheng H. (2023). Cycloheximide (CHX) Chase Assay to Examine Protein Half-life. Bio. Protoc..

[32] Paul A., Gunewardena S., Stecklein S.R., Saha B., Parelkar N., Danley M., Rajendran G., Home P., Ray S., Jokar I. (2014). PKC*λ*/*ι* signaling promotes triple-negative breast cancer growth and metastasis. Cell Death Differ..

[33] Towbin H., Staehelin T., Gordon J. (1979). Electrophoretic transfer of proteins from polyacrylamide gels to nitrocellulose sheets: Procedure and some applications. Proc. Natl. Acad. Sci. USA.

[34] Schindelin J., Arganda-Carreras I., Frise E., Kaynig V., Longair M., Pietzsch T., Preibisch S., Rueden C., Saalfeld S., Schmid B. (2012). Fiji: An open-source platform for biological-image analysis. Nat. Methods.

[35] Shihan M.H., Novo S.G., Le Marchand S.J., Wang Y., Duncan M.K. (2021). A simple method for quantitating confocal fluorescent images. Biochem. Biophys. Rep..

